# Videoconference-Based Physical Performance Tests: Reliability and Feasibility Study

**DOI:** 10.3390/ijerph19127109

**Published:** 2022-06-09

**Authors:** Ander Espin, Julia García-García, Unai Latorre Erezuma, Maialen Aiestaran, Jon Irazusta, Ana Rodriguez-Larrad

**Affiliations:** 1 Ageing on Research Group, Department of Physiology, University of the Basque Country (UPV/EHU), 48940 Leioa, Spain; ander.espin@ehu.eus (A.E.); julia.garcia@ehu.eus (J.G.-G.); unai.latorre@ehu.eus (U.L.E.); maialen.aiestaran@ehu.eus (M.A.); ana.rodriguez@ehu.eus (A.R.-L.); 2 Clinical Nursing and Community Health Research Group, Biocruces Bizkaia Health Research Institute, 48903 Barakaldo, Spain

**Keywords:** fitness test, tele-assessment, tele-health, intraclass correlation coefficient, standard error of measurement

## Abstract

Validated tools to evaluate physical performance remotely with real-time supervision are lacking. We assessed test–retest and inter-rater reliability, as well as the feasibility of carrying out the five-repetition sit-to-stand (5RSTS), kneeling push-up (KPU) and Shirado–Ito trunk flexor endurance (SIF) tests by 1:1 real-time videoconference. We also evaluated the correlation of these tests with measures of self-reported physical fitness, physical activity, health state and pain. A total of 96 healthy adults participated in the study (18–65 years). Relative and absolute reliabilities were assessed with the intraclass correlation coefficient (ICC) and standard error of measurement (SEM), respectively. Feasibility outcomes included testing duration, participant acceptability (1–5 Likert scale) and presence of adverse events. Self-reported measures were obtained with validated online questionnaires, and correlations were analyzed with Pearson’s partial correlation coefficients controlling for age. ICCs were excellent (>0.9), and SEMs were generally low (2.43–16.21%). The mean duration of all tests was <5 min, mean acceptability was ≥4.5, and adverse events were few. The KPU showed statistically significant correlations with various self-reported measures (*p* < 0.05). In conclusion, the 5RSTS, KPU and SIF were reliable and feasible when conducted by 1:1 real-time videoconference. This study provides a tool that could be logistically and economically advantageous.

## 1. Introduction

The outbreak of the COVID-19 pandemic made tele-health solutions indispensable for continuation of healthcare service while avoiding physical contact [1]. Due to its advantages and opportunities in the current global situation, tele-health has been proposed as an alternative method for physical assessments and exercise programs [2]. For some authors, the transition to tele-health is considered a positive change that should be maintained in the post-pandemic “new normal” [3]. However, this transition requires that provision of tele-health services be robust and reliable.

Measuring the physical performance of individuals provides vital information about overall health and wellness. In addition, physical performance tests are a crucial element to assess the effects of exercise interventions. Therefore, those tests are a key component of tele-health assessments and exercise programs. Although apps to measure physical capacity are increasingly being developed [4], there is still a lack of validated tools to evaluate physical performance remotely with real-time supervision. Some of the exercise programs delivered remotely due to COVID-19 adapted their physical assessments so that they could be carried out online [5,6]. However, important modifications were made to the original testing protocols that could bias the test results. For example, Jennings et al., replaced a dumbbell with a water gallon or canvas bag [5], and Gonzalez-Gerez et al., described a home-space test that usually requires a 30 m course [6]. In addition, the reliability and validity of these tests were not assessed previously. Other studies of online exercise programs opted to maintain physical assessments in the traditional face-to-face manner when allowed [7]. Others had to rely on subjective measures based on self-perception to evaluate changes in physical fitness [8], and finally, other studies such as the one by Costa et al., did not include any fitness tests to analyze the effectiveness of their exercise interventions [9].

Due to the COVID-19 pandemic, some authors proposed equipment-free and easy-to-administer tests to replace face-to-face evaluations with tele-assessments [10,11]. However, the remote-delivery versions of many of these tools have not been validated, and future research should focus on filling the gap related to the analysis of feasibility and measurement characteristics of remote physical assessments [10].

To date, the few studies analyzing the validity and reliability of videoconference-based physical assessments have focused on specific tests for people with specific musculoskeletal conditions or pathologies, such as knee arthroplasty patients [12]. Furthermore, most have included small samples of participants and raters and have been identified as having a high risk of bias [12]. To our knowledge, no previous study has assessed the reliability of real-time videoconference-based tests to evaluate physical performance in the general healthy population, and high-quality research is needed to fill this gap.

Therefore, the aim of this study was to assess the test–retest and inter-rater reliability, as well as the feasibility, of three different physical performance tests carried out by 1:1 real-time videoconference in a large sample of healthy adults. We established that the tests had to: (a) assess upper and lower limb and trunk performance; (b) be equipment-free and quick and easy to administer; and (c) be appropriate for the entire adult population (18–65 years). Therefore, we selected the following previously validated tests: the five-repetition sit-to-stand test (5RSTS) [13] (lower limb strength), the kneeling push-up test (KPU) [14] (upper limb strength) and the Shirado–Ito trunk flexor endurance test (SIF) [15] (trunk flexor endurance). As a secondary aim, we analyzed the correlation of test performance with participants’ self-reported physical fitness, physical activity, health state and pain. We hypothesized that the three performance tests would be reliable and feasible when conducted by real-time videoconference, and that they would significantly correlate with self-reported physical fitness, physical activity, health state and pain.

## 2. Materials and Methods

### 2.1. Participants

A total of 96 healthy adults participated in the study: 64 (32 women and 32 men) in the test–retest reliability analysis and 32 (16 women and 16 men) in the inter-rater reliability analysis. In each analysis and within each sex, participants were evenly distributed in 4 age ranges: 18–29 years, 30–39 years, 40–49 years and 50–65 years. Sampling was performed based on the guidelines by Koo and Li [16], which proposed heterogeneous samples of at least 30 participants as a rule of thumb in reliability studies. This allowed us to analyze the test–retest reliability separately in each sex and assess inter-rater reliability independently. Inclusion criteria were being between 18 and 65 years old and having a laptop with a camera and internet access. The only exclusion criterion was having a musculoskeletal condition that would not allow test performance. Participants were recruited by snowball sampling and gave informed written consent before participating in the study, which was approved by the Ethics Committee for Research Involving Human Beings of the University of the Basque Country (M10/2020/324).

### 2.2. Questionnaires

Prior to physical performance testing, participants responded to an online questionnaire that collected the following information:

#### 2.2.1. Sociodemographic Data

Age, sex, height, mass, socio-professional status (studying/working or unemployed/retired), videoconferencing habit (yes/no response to the question “are you, because of your work, studies or other reasons, used to using videoconferencing systems regularly?”) and bodyweight strength training habit (yes/no response to the question “do you perform muscle strengthening exercises such as squats, push-ups or abdominal crunches regularly?”).

#### 2.2.2. Self-Reported Physical Fitness

The International Fitness Scale (IFIS) was used [17]. It consists of 5 items with which participants rate their own general physical fitness, cardiorespiratory fitness, muscular strength, speed/agility and flexibility in a 5-point Likert scale ranging from 1 (very poor) to 5 (very good).

#### 2.2.3. Physical Activity

The 8-response single-response item of physical activity (PA8) was used [18]. Participants made a single selection from 8 potential physical activity descriptors ranging from 1 (I do not exercise/walk regularly now and I do not intend to start in the near future) to 8 (I have been doing moderate physical activity 5 or more days a week or vigorous activity at least 3 days a week, for 7 months or longer).

#### 2.2.4. Health State

The 0–100 scale from the EuroQol-5D questionnaire was used [19]. Participants gave a value ranging from 0 (worst imaginable health state) to 100 (best imaginable health state) to their current health state.

#### 2.2.5. Pain

The pain domain from the Short-Form 36 (SF36) health survey was used [20]. It consists of 2 Likert-response items regarding pain intensity and interference with work over the last 4 weeks. From the answers to both items, a score ranging from 0 to 100 is obtained, with a lower score indicating higher pain.

### 2.3. Testing Procedure

**Figure 1:**
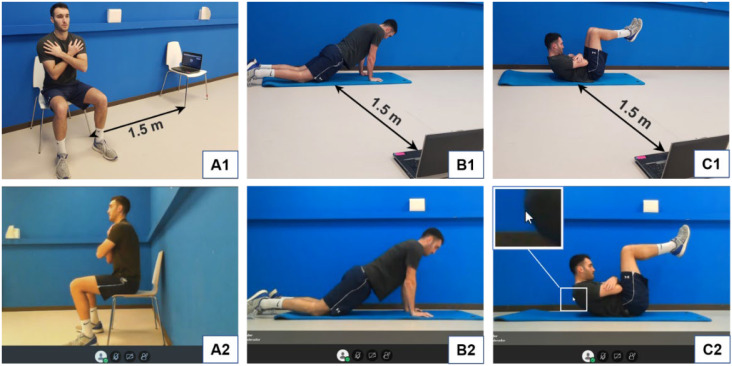
Participant performing the 5-repetition sit-to-stand test (**A1**,**A2**), the kneeling push-up test (**B1**,**B2**) and the Shirado-Ito trunk flexor endurance test (**C1**,**C2**). (**A1**–**C1**) show the positioning of the participant and the material. (**A2**–**C2**) show the screen view of the evaluator. In (**C2**), the evaluator is using the mouse cursor to accurately assess the position of the trunk.

The physical performance tests were carried out by real-time 1:1 videoconference using the Blackboard Collaborate system (Blackboard Inc., Washington, DC, USA), and audio and video were continuously shared between evaluator and participant. To access the videoconference, participants only had to enter a previously received link in their web browser and allow the system to share audio and video. Participants were given written instructions with images about how to prepare the space for the tests. For the 5RSTS, they had to place a chair against a wall or similar surface and put the laptop on another chair of the same height 1.5 m away, so that a side view was obtained (Figure 1(A1)). For the KPU and SIF, participants had to place their laptop and a mat on the floor, with a distance of 1.5 m and obtaining a side view again (Figure 1(B1,C1)). Participants were instructed to wear comfortable sportswear that did not limit movement, a tight shirt and sneakers, and they had to tie their hair if it was long, with the aim of allowing precise anatomical reference identification. They were asked to avoid strenuous physical activity in the 24 h before the tests. Tests were always conducted in the same order: 5RSTS, KPU and SIF. This order was established based on the physical fatigue generated by the tests, from lowest to highest, with the aim that the performance of each test had the least possible influence on the execution of the following one. Each test was first explained and executed as an example by the evaluator and subsequently performed by the participant. Participants were allowed 2–3 min rest between tests, coinciding with the evaluator’s explanations of the following test. The level of fatigue for each test and the necessary rest time between the tests were determined based on the subjective perceptions of participants in pilot tests prior to conducting the study. No warm-up was performed before starting the tests. Participants were asked to give their maximum effort in each test but were not verbally encouraged during test execution.

#### 2.3.1. 5-Repetition Sit-to-Stand Test (5RSTS)

This test consists of standing up from and sitting down on a chair 5 times as quickly as possible [13]. Participants were asked to sit on the front half of the seat, back straight and arms crossed over their chest. Feet were placed flat on the ground, shoulder width apart and slightly back from the knees (Figure 1(A1,A2)). The 5 sit-to-stand repetitions were manually timed with a digital stopwatch. The count started when the participant’s buttocks left the seat and ended when they contacted the seat again after the fifth repetition. Participants had to fully stand to complete hip extension, and just touching the chair with their buttocks was enough when sitting. At least one training trial was performed before beginning the test. After that, participants performed the test twice, with 30 s rest between trials, and the mean duration was registered in seconds and hundredths. Participants used their own chair, which, in all cases, had a firm (not padded) seat and no armrests. Participants were asked to use a chair with a seat height of approximately 43 cm. However, not having a chair of that exact height was not an exclusion criterion. The height of the chair of all the participants was registered, and the average was 44.6 ± 1.8 cm.

#### 2.3.2. Kneeling Push-Up Test (KPU)

This test consists of performing the maximum number of push-ups possible [14]. Participants started lying face-down on a mat or similar surface, hands pointing forward under the shoulders and ankles plantar-flexed. Using their knees as the pivot point, participants had to raise their body until full elbow extension (Figure 1(B1,B2)), then lower themselves until touching the mat with their nose. Their stomach could not touch the mat when returning down, and no instructions were given regarding execution speed. One or two training repetitions were performed before starting the test, which was performed only once. The total number of correct repetitions performed consecutively without rest was registered. The first repetition was recorded when the participants raised their body for the second time, and a new repetition was subsequently counted every time they returned to the up position.

#### 2.3.3. Shirado–Ito Trunk Flexor Endurance Test (SIF)

This test consists of maintaining a defined trunk flexion position for as long as possible [15]. Participants started lying on the mat in a supine position, hips and knees flexed at 90°, hands grasping the opposite arm and forearms resting on the body. From this position, participants flexed their trunk maximally, while maintaining maximum flexion of their cervical spine. To accurately assess the position of the trunk, the evaluator used the mouse cursor on the screen. First, when the participant was completely lying flat, the mouse cursor was placed on the most cranial point of the back that was in contact with the mat (i.e., the point in which the curvature of the shoulder starts). Then, when the participant flexed the trunk, the evaluator waited for 5 s so that a stable position was achieved, and immediately raised the mouse cursor vertically until it “contacted” the participant’s back (Figure 1(C2)). Unlimited warnings were given to the participant every time their back fell below the mouse cursor due to trunk flexor fatigue. Hip and knee angles were also verbally corrected when necessary. The test finished when the participant stopped or was unable to regain the starting position despite the evaluator’s warnings. After each warning, participants were asked to immediately flex their trunk, and the test was stopped if they did not regain the starting position in the following 2 s. Participants tried the position before starting the test, which was performed only once, and the total time was registered in seconds.

#### 2.3.4. Test–Retest Reliability

To assess test–retest reliability, the 3 tests were repeated 7 days later at a similar time of the day. These tests were always conducted by the same evaluator, who was located in a prepared laboratory, using a laptop with an optical fiber wired Ethernet connection (maximum theoretical speed: symmetrical 1 Gbps), while participants were located in their home (89%) or workplace (11%), using a laptop with Wi-Fi connection.

#### 2.3.5. Inter-Rater Reliability

To assess inter-rater reliability, 4 evaluators were included in the study (evaluator from test–retest analysis + 3 others). The number of evaluators was established based on the guidelines by Koo and Li [16], who recommend that at least 3 raters should be involved in reliability studies. The additional 3 evaluators received a 3 h session to familiarize them with the test protocols and evaluation criteria. Each evaluator assessed 8 participants once. These assessments were recorded and subsequently shared with the other evaluators, so that they could analyze them too. Evaluators were blinded to each other’s ratings, and they played each recording only once to simulate as well as possible a real-time assessment. To avoid possible biases in the SIF, participants in the inter-rater reliability analysis continued receiving warnings even if they were not able to regain the starting trunk position above the mouse cursor, until they stopped the test on their own. In these assessments, evaluators were located in their home (69%) or workplace (31%), using their personal laptops with Wi-Fi connection (evaluators 1–4, in order, maximum theoretical speed: 1 Gbps, 600 Mbps, 600 Mbps, 1 Gbps), while participants were situated in their home (81%) or workplace (19%), using a laptop with Wi-Fi connection.

### 2.4. Feasibility Outcomes

#### 2.4.1. Testing Duration

The duration of each individual test was registered in minutes. The beginning of each test was set when the evaluator began their explanations, and the end of the test was established when the participant finished executing it. The total testing duration was calculated as the sum of the 3 individual test durations.

#### 2.4.2. Participant Acceptability

Acceptability was evaluated with 6 sentences with which participants had to report their agreement level in a 5-point Likert scale ranging from 1 (totally disagree) to 5 (totally agree). The topics covered by these sentences were (a) videoconferencing system: “the system used for videoconferencing is easy to use”, (b) communication quality: “communication with the evaluator has been satisfactory”, (c) resource preparation: “it has been easy to prepare the space to carry out the tests”, (d) easiness of the tests: “the tests used are easy to understand and perform”, (e) duration of the tests: “the time taken to carry out the tests has been reasonable” and (f) general feasibility: “in general, I think it is feasible to carry out these tests by videoconference”. This information was collected by an interview just after finishing the tests on the first testing day, and when participant agreement was not maximum (i.e., 5/5), they were asked to give the reason.

#### 2.4.3. Adverse Events

Adverse events were recorded on the first testing day and were divided into 2 types: (a) technical (connection and/or operation problems with the videoconferencing system) and (b) participant safety-related (pain, discomfort, or any other health-related problem that appeared during the tests). They were also classified as minor (those that slightly hindered test development) and major (those that prevented test development). Additionally, appearance of delayed onset muscle soreness (DOMS), as well as its location and days of duration were collected.

### 2.5. Statistical Analysis

Continuous data are shown using mean ± standard deviation, while categorical data are presented as frequency and percentage. For continuous data, the normality of distribution was checked with the Shapiro–Wilk test. Data with non-normal distribution were square root-transformed for statistical analyses. To assess relative reliability, the intraclass correlation coefficient (ICC) and its 95% confidence interval (CI) were used, based on a single-rating, absolute agreement, two-way mixed-effects model for the test–retest analysis and single-rating, absolute agreement, two-way random-effects model for the inter-rater analysis [16]. The ICC values were categorized as poor (<0.5), moderate (0.05–0.75), good (0.75–0.9) and excellent (>0.9) [21]. To assess absolute reliability, the standard error of measurement (SEM) was used, calculated as the square root of the mean square error term in a repeated measures ANOVA [22]. Additionally, the coefficient of variation of SEM was calculated by obtaining its percentage to the mean test value (mean of the test and retest or mean of the 4 evaluators’ ratings). Student’s paired samples *t* test was used to analyze differences between test and retest to detect possible learning effects. Differences between women and men in feasibility outcomes were analyzed with Student’s independent samples T test for continuous variables and the Chi-squared test for categorical variables. The correlation of the tests with self-reported physical fitness, physical activity, health state and pain was assessed using Pearson’s partial correlation coefficient (r) controlling for age. For these correlations, the mean value between test and retest or the mean value between the 4 evaluators’ ratings was used. The absolute r values were categorized as negligible (0.0–0.3), low (0.3–0.5), moderate (0.5–0.7), high (0.7–0.9) and very high (>0.9) [23]. All analyses were performed separately for each sex, except for the inter-rater reliability. The significance level was set at *p* < 0.05. Statistical analysis was performed using IBM SPSS Statistics for Windows version 24.0 (IBM Corp., Armonk, NY, USA).

## 3. Results

### 3.1. Participants

Descriptive data of the participants are shown separately in different groups depending on the type of reliability analysis (test–retest and inter-rater) and sex (women and men) and can be found in Table 1.

### 3.2. Test–Retest Reliability

Main test–retest reliability measures are shown in Table 2. No statistically significant differences were observed between test and retest (*p* > 0.05).

### 3.3. Inter-Rater Reliability

Main inter-rater reliability measures are shown in Table 3.

### 3.4. Feasibility

Feasibility measures are shown in Table 4. In both sexes, the most frequent reasons for not giving the maximum agreement score were (a) videoconferencing system: difficulties with setting up the computer to share audio and video, (b) communication quality: low quality or lagged audio, (c) resource preparation: lack of space, (d) easiness of the tests: technical complexity or too high physical demand in the KPU, (e) duration of the tests: excessive time in test explanations and (f) general feasibility: loss of information or reduced communication capacity because the tests were not conducted face-to-face. There were no major adverse events. Women experienced a higher rate of minor technical adverse events (*p* = 0.036). In both sexes, most frequent technical adverse events were occasional communication disruptions and video freezes due to deficient internet connection, while the most frequent participant safety-related adverse event was slight discomfort in the cervical or lumbar region in the SIF. Most common DOMS location was the upper limb (45.8% in women and 43.8% in men), followed by the trunk (8.3% in women and 6.3% in men) and the lower limb (2.1% in both women and men).

### 3.5. Correlations between Physical Performance Test Results and Questionnaires

Pearson’s partial correlations between the physical performance test results and the questionnaire scores are shown in Table 5. Positive and statistically significant correlations were found between the number of KPU repetitions and self-reported general physical fitness (*p* = 0.004), muscular strength (*p* < 0.001) and speed/agility (*p* = 0.038) in women and self-reported general physical fitness (*p* = 0.002), cardiorespiratory fitness (*p* = 0.001), muscular strength (*p* = 0.002), physical activity (*p* < 0.001) and health state (*p* = 0.023) in men. No statistically significant correlations were observed between 5RSTS or SIF performance and any of the questionnaires (*p* > 0.05).

## 4. Discussion

The main objective of this study was to assess the reliability and feasibility of carrying out three physical performance tests by 1:1 real-time videoconference. The tests analyzed were the 5RSTS, the KPU and the SIF, which are all equipment-free, quick and easy to administer and allow the assessment of all main muscle groups in the whole adult population. Test–retest and inter-rater reliability, as well as feasibility, were excellent for the three tests, confirming that they can be validly carried out by real-time videoconference. To our knowledge, this is the first study exploring the reliability and feasibility of physical performance tests carried out by real-time videoconference in a large sample of healthy adults. This study provides a tool that can be both logistically and economically advantageous in research, clinical or fitness settings, as time and money costs associated with displacements are avoided [24]. Moreover, these tests are compatible with situations in which interpersonal physical distancing is required, such as the current global COVID-19 pandemic and other possible similar future scenarios.

### 4.1. Reliability: Comparisons with Previous Studies

The relative 5RSTS test–retest reliability found in our study (ICC = 0.92 in women and ICC = 0.98 in men) is very similar to that found by Bohannon et al. [25] (ICC = 0.975) and Staartjes and Schröder [26] (ICC = 0.96), who performed this test in the traditional face-to-face modality in the general healthy population. Our absolute test–retest reliability (SEM = 4.32% in women and SEM = 2.95% in men) is also very close to that obtained by Bohannon et al., (SEM = 4.5%). The relative inter-rater reliability observed in our sample with the 5RSTS (ICC = 0.99) is slightly higher than that found by Simmonds et al. [27] (ICC = 0.91) in the face-to-face modality in healthy adults. Our absolute inter-rater reliability (SEM = 2.43%) with this test is also very close to that obtained by Simmonds et al., (SEM = 3%).

With the KPU, we obtained a higher relative test–retest reliability (ICC = 0.98 in women and ICC = 0.96 in men) than Wood and Baumgartner [28] (ICC = 0.83), who performed this test in the traditional face-to-face modality in college-age women. This difference may be due to the fact that the sample used by Wood and Baumgartner was more homogeneous than ours, as the participants were all young and physically active women with less variable performances, which could lead to a lower ICC [16]. Our relative inter-rater reliability (ICC = 0.96) is very similar to that of Wood and Baumgartner (ICC = 0.997). To our knowledge, our study is the first to report the absolute test–retest and inter-rater reliabilities of the KPU. We found test–retest SEMs of 16.21% in women and 9.94% in men and an inter-rater SEM of 5.80%.

Finally, we observed an excellent relative test–retest reliability with the SIF (ICC = 0.93 in both women and men). When analyzing the relative test–retest reliability of the SIF carried out face-to-face, Ito et al., found an ICC of 0.95 in healthy adults [15], del Pozo-Cruz et al., found ICCs of 0.96 and 0.97 in female and male adult low back pain patients [29], and Juan-Recio et al., found an ICC of 0.80 in young and physically active men [30]. Our test–retest ICCs are very close to those of Ito et al., and del Pozo Cruz et al., but are higher than that obtained by Juan-Recio et al. The reason for this difference could be that the sample used by Juan-Recio et al., was more homogeneous than ours [16]. The absolute test–retest reliability of our study (SEM = 10.61% in women and SEM = 12.03% in men) is lower than that found by del Pozo-Cruz et al., (SEM = 3.40% in women and 4.70% in men). However, Juan-Recio et al., obtained a typical error (an absolute reliability measure very similar to SEM) of 19.89%. To our knowledge, our study is the first to report inter-rater reliability of the SIF. We found excellent relative (ICC = 0.97) and absolute (SEM = 5.64%) inter-rater reliabilities.

### 4.2. Feasibility

The duration of all tests was very short, supporting their feasibility in the videoconference format. Each test lasted fewer than 5 min on average, and the total mean duration was of little more than 10 min. To our knowledge, this is the first study reporting the duration (including evaluator explanations and participant execution) of the 5RSTS, KPU and SIF in healthy adults. Acceptability was very high, with all assessed topics obtaining mean scores of 4.5 or higher. The aspect with the lowest acceptability was general feasibility. The main reason was that although participants considered the tests to be feasible when conducted by videoconference, they preferred them to be conducted in person. Finally, there were no major adverse events, and minor adverse events were few. Most technical adverse events were due to a deficient internet connection, so ensuring a high-quality connection is key for satisfactory test development. The most common participant safety-related adverse event was mild discomfort in the spine during the SIF. However, this does not seem to be exclusive to the videoconference modality, as a previous study found that over 20% of SIFs were stopped due to spinal pain when performed in the traditional face-to-face manner [31]. Almost half of the participants suffered from DOMS. To our knowledge, this is the first study reporting DOMS caused by the 5RSTS, KPU and SIF in healthy adults.

### 4.3. Correlations between Muscle Performance Test Results and Questionnaires

The number of push-up repetitions was significantly and positively associated with IFIS, PA8 and EuroQol-5D test scores. Therefore, the videoconference-based KPU appears to be a good indicator of fitness, physical activity and health in the adult population. However, no significant correlations were observed between the questionnaire results and the 5RSTS and SIF. It is possible that some individual anthropometric characteristics, such as height [32] and mass or lower trunk width [30], which have been demonstrated to directly influence the results of the 5RSTS [32] and SIF [30], acted as confounding factors and prevented significant correlations. Moreover, some authors have suggested that the 5RSTS could have a ceiling effect in healthy and physically well-functioning adults [33]. Finally, none of the tests showed significant correlation with pain. Other authors have observed that higher 5RSTS times [26,27] and lower SIF times [29] are significantly correlated with increased low back pain [26,27] and disability [26,27,29]. However, all these studies included low back pain patients. On the contrary, the participants in our sample were healthy, and generally, the few people who had pain had low-intensity pain with little or no interference with daily activities.

### 4.4. Limitations

Most of the study participants were used to using videoconferencing systems, and approximately half of them regularly performed bodyweight strengthening exercises similar to those used in the tests. These two points could have led to higher reliability and feasibility results, which may not be transferable to other populations with less knowledge about computer science and bodyweight training. Similarly, the videoconference modality makes the tests presented in this study depend on internet access, so they may not be applicable in certain populations from rural areas. In addition, although participants were verbally prompted to give maximal effort, this could not be objectively controlled. Finally, within the test–retest analysis, the questionnaires with self-reported measures were only performed on the first testing day, as we considered that those measures would hardly vary significantly in a period of 7 days. However, it is possible that changes in any of the measures could have slightly influenced the execution on retest.

Future studies should include reliability analyses focused on other populations, such as the elderly, as well as on people who are not used to videoconferencing systems and muscle strengthening exercises. Performing similar reliability and feasibility studies on tests that assess fitness components not evaluated in this study, such as cardiorespiratory function, could also be beneficial.

## 5. Conclusions

The 5RSTS, KPU and SIF are reliable and feasible when conducted by 1:1 real-time videoconference in healthy adults. The tests are rapid, performed without equipment, and valid for assessing all main muscle groups in the whole adult population. This study provides a tool that could be logistically and economically advantageous in research, clinical or fitness settings, and is compatible with situations in which interpersonal physical distancing is required.

## Figures and Tables

**Table 1:** Descriptive data of the participants.

Variable	Test–Retest Reliability Analysis		Inter-Rater Reliability Analysis	
	Women (*n* = 32)	Men (*n* = 32)	Women (*n* = 16)	Men (*n* = 16)
Sociodemographic data				
Age (years)	40.7 ± 12.4	40.4 ± 12.2	40.8 ± 13.6	39.2 ± 14.3
Height (cm)	163.8 ± 5.0	177.9 ± 5.6	163.5 ± 4.3	178.1 ± 7.6
Weight (kg)	59.6 ± 6.3	76.3 ± 8.9	61.4 ± 8.7	77.9 ± 11.7
Body Mass Index (kg/m^2^)	22.2 ± 2.1	24.1 ± 2.6	23.0 ± 2.8	24.5 ± 2.9
Socio-professional status				
Studying, *n* (%)	2 (6.3)	2 (6.3)	0 (0.0)	3 (18.8)
Working or unemployed, *n* (%)	29 (90.6)	29 (90.6)	16 (100.0)	13 (81.3)
Retired, *n* (%)	1 (3.1)	1 (3.1)	0 (0.0)	0 (0.0)
Videoconferencing habit, *n* (%)	28 (87.5)	21 (65.6)	11 (68.8)	12 (75.0)
Muscle strengthening habit, *n* (%)	15 (46.9)	19 (59.4)	8 (50.0)	7 (43.8)
International Fitness Scale (1–5)				
General fitness	3.3 ± 1.0	3.7 ± 0.7	3.4 ± 0.7	3.6 ± 0.8
Cardiorespiratory fitness	3.3 ± 0.9	3.8 ± 0.5	3.3 ± 0.8	3.8 ± 0.8
Muscular strength	3.2 ± 0.8	3.6 ± 0.7	2.9 ± 0.9	3.5 ± 0.7
Speed/Agility	3.3 ± 0.6	3.6 ± 0.7	3.5 ± 0.7	3.6 ± 0.6
Flexibility	3.3 ± 0.9	2.5 ± 1.0	2.6 ± 1.2	3.3 ± 1.1
PA8 physical activity (1–8)	4.9 ± 1.8	6.1 ± 1.9	4.4 ± 1.1	5.3 ± 1.8
EuroQol-5D health state (0–100)	75.8 ± 14.8	76.6 ± 12.2	77.1 ± 10.0	73.8 ± 15.5
SF36 pain (0–100)	85.1 ± 15.9	84.0 ± 19.7	77.6 ± 17.6	82.8 ± 15.4

**Table 2:** Test–retest reliability in women (*n* = 32) and men (*n* = 32).

Variable	Test	Retest	ICC (95% CI)	SEM (%)
5RSTS (seconds)				
Women	6.31 ± 1.05	6.19 ± 1.03	0.92 (0.85–0.96)	0.27 (4.32)
Men	6.13 ± 1.29	6.08 ± 1.32	0.98 (0.96–0.99)	0.18 (2.95)
KPU (repetitions)				
Women	11.63 ± 9.29	12.06 ± 9.02	0.98 (0.96–0.99)	1.92 (16.21)
Men	33.84 ± 15.99	34.38 ± 16.65	0.96 (0.92–0.98)	3.39 (9.94)
SIF (seconds)				
Women	77.88 ± 33.21	77.03 ± 34.32	0.93 (0.86–0.97)	8.22 (10.61)
Men	69.03 ± 33.55	66.63 ± 30.71	0.93 (0.87–0.97)	8.16 (12.03)

Note: % in SEM refers to the coefficient of variation (i.e., percentage to the mean test value). Abbreviations: CI = confidence interval; ICC = intraclass correlation coefficient; KPU = kneeling push-up test; SEM = standard error of measurement; SIF = Shirado–Ito trunk flexor endurance test; 5RSTS = 5-repetition sit-to-stand test.

**Table 3:** Inter-rater reliability (*n* = 32).

Variable	Rater 1	Rater 2	Rater 3	Rater 4	ICC (95% CI)	SEM (%)
5RSTS (s)	6.31 ± 1.67	6.21 ± 1.76	6.30 ± 1.74	6.12 ± 1.72	0.99 (0.98–1.00)	0.15 (2.43)
KPU (r)	20.50 ± 19.17	20.81 ± 18.85	19.38 ± 19.42	20.91 ± 18.82	0.96 (0.93–0.98)	1.18 (5.80)
SIF (s)	69.94 ± 38.40	69.41 ± 36.56	67.55 ± 36.49	68.95 ± 36.83	0.97 (0.94–0.98)	3.89 (5.64)

Note: % in SEM refers to the coefficient of variation (i.e., percentage to the mean test value). Abbreviations: CI = confidence interval; ICC = intraclass correlation coefficient; KPU = kneeling push-up test; r = repetitions; s = seconds; SEM = standard error of measurement; SIF = Shirado–Ito trunk flexor endurance test; 5RSTS = 5-repetition sit-to-stand test.

**Table 4:** Summary of feasibility outcomes.

Variable	Women (*n* = 48)	Men (*n* = 48)
Testing duration (minutes)		
5RSTS	3.9 ± 1.2 (2.1–8.0)	3.6 ± 1.3 (1.2–7.0)
KPU	4.3 ± 1.1 (2.3–7.0)	4.0 ± 1.2 (2.6–8.0)
SIF	4.4 ± 1.3 (2.0–8.8)	4.2 ± 1.5 (2.0–10.0)
Total	12.7 ± 2.6 (7.9–19.5)	11.9 ± 2.9 (8.0–20.0)
Participant acceptability (1–5)		
Videoconferencing system	4.9 ± 0.4 (3.0–5.0)	4.9 ± 0.5 (2.0–5.0)
Communication quality	4.8 ± 0.5 (3.0–5.0)	4.9 ± 0.2 (4.0–5.0)
Resource preparation	4.7 ± 0.6 (3.0–5.0)	4.8 ± 0.4 (4.0–5.0)
Easiness of the tests	4.8 ± 0.5 (3.0–5.0)	4.8 ± 0.5 (2.0–5.0)
Duration of the tests	5.0 ± 0.3 (3.0–5.0)	5.0 ± 0.2 (4.0–5.0)
General feasibility	4.7 ± 0.6 (3.0–5.0)	4.5 ± 0.8 (1.0–5.0)
Adverse events, *n* (%)		
Technical, minor	13 (27.1)	5 (10.4) *
Participant safety, minor	6 (12.5)	4 (8.3)
DOMS appearance	25 (52.1)	22 (45.8)
DOMS duration days	2.3 ± 1.0 (1.0–6.0)	2.6 ± 1.5 (1.0–7.0)

Notes: data between brackets refer to the minimum and maximum values; there were not major adverse events. * Statistically significant difference between women and men (*p* < 0.05 in Chi squared test). Abbreviations: DOMS = delayed-onset muscle soreness; KPU = kneeling push-up test; SIF = Shirado–Ito trunk flexor endurance test; 5RSTS = 5-repetition sit-to-stand test.

**Table 5:** Pearson’s partial correlation coefficients (r) between physical performance test results and questionnaire scores controlling for age in women (*n* = 48) and men (*n* = 48).

	IFIS General (1–5)	IFIS Cardio (1–5)	IFIS Strength (1–5)	IFIS Speed (1–5)	IFIS Flexibility (1–5)	PA8 (1–8)	EuroQol-5D (0–100)	SF36 Pain (0–100)
5RSTS (s)								
Women	0.030	0.037	−0.172	−0.179	0.095	−0.015	−0.027	0.104
Men	−0.187	−0.243	−0.056	−0.031	−0.073	−0.268	−0.129	0.142
KPU (r)								
Women	0.415 **	0.224	0.514 ***	0.303 *	0.038	0.235	0.142	0.152
Men	0.442 **	0.478 **	0.435 **	0.186	0.140	0.554 ***	0.332 *	0.014
SIF (s)								
Women	0.104	0.155	0.280	0.060	0.161	−0.156	−0.039	−0.061
Men	−0.039	0.129	0.114	0.009	−0.204	−0.064	−0.049	0.048

* *p* < 0.05, ** *p* < 0.01, *** *p* < 0.001. Abbreviations: IFIS = International Fitness Scale; KPU = kneeling push-up test; PA8 = 8-response single-response item of physical activity; r = repetitions; s = seconds; SIF = Shirado–Ito trunk flexor endurance test; 5RSTS = 5-repetition sit-to-stand test.

## Data Availability

The data that support the findings of this study are available from the corresponding author upon reasonable request.
